# Editorial: Parasites and Cancer

**DOI:** 10.3389/fmed.2019.00055

**Published:** 2019-03-22

**Authors:** Monica C. Botelho, Joachim Richter

**Affiliations:** ^1^Department of Health Promotion and Chronic Diseases, INSA, National Institute of Health Dr. Ricardo Jorge, Porto, Portugal; ^2^I3S, Instituto de Investigação e Inovação da Universidade do Porto, Porto, Portugal; ^3^Institute of Tropical Medicine and International Health, Charité University Medicine, Berlin, Germany

**Keywords:** Cancer-associated parasites, *Schistosoma haematobium*, *Echicococcus granulosus*, *Theileria*, *Fasciola hepatica*, *Opisthorchis viverrini*, bladder cancer, cholangiocarcinoma

Emerging evidence indicates that certain parasites such as the blood fluke *Schistosoma haematobium*, and small liver flukes *Opisthorchis viverrini* and *Clonorchis sinensis* are causative agents of malignancies such as bladder cancer caused bv schistosomes and cholangiocarcinoma by liver flukes. In many endemic regions these helminths are responsible for the majority of cancer cases. Parasites, other than helminths, are also associated with cancers, such as *Theileria*, an intracellular eukaryotic parasite. On the contrary, some parasite infections or molecules seem to display protective effects on some cancers, such as is the case with *Echinococcus*.

Therefore, understanding how these parasites cause/promote or hinder oncogenesis in humans will aid to develop novel strategies for controlling the parasitosis and for preventing and treating the infection-associated malignancy.

The Infectious Diseases—Surveillance, Prevention, and Treatment section of the journal Frontiers in Medicine, in partnership with the journal Frontiers in Public Health, hosted the first Research Topic on Parasites and Cancer with the aim to facilitate global parasites infection-associated cancer elimination through scientific advances.

Nearly 40 authors, representatives from Australia, Brazil, Ethiopia, Germany, Portugal, United States, and Thailand, participated in this Research Topic covering all continents of the world. Some of these authors are the most cited in the field of parasites and cancer: Ross H. Andrews, Paul Brindley, Michael H. Hsieh, Alex Loukas, Donald McManus, Trevor N. Petney, Paiboon Sithithaworn, and Puangrat Yongvanit.

This Research Topic comprises eight papers, spanning topics that cover epidemiology, evolution of helminths, mechanisms of parasite-associated oncogenesis and potential therapeutics, diagnostics, host pathology, parasite biology, health burden, and prevention and control programs. Michael J. Smout from James Cook University (Townsend, Australia) presented a work on angiogenesis induced by granulin, a molecule secreted by the liver-fluke *Opistorchis viverrini* (Smout et al). Bayssa Chala (Adama Science and Technology University, Adama, Ethiopia) and Workineh Torben (Tulane National Primate Research Center, Covington, LA, United States) wrote about the epidemiology of urogenital schistosomiasis in Ethiopia (Chala and Torben).

Shiwanthi L. Ranasinghe and Donald P. McManus (QIMR Berghofer Medical Research Institute, Brisbane, QLD, Australia) introduced a very interesting topic: the use of heminth-derived molecules that might either indirectly help controlling tumors through the immune response they induce, or display a direct effect on tumor cells. In this case the use of proteins from *Echinococcus granulosus* is described (Ranasinghe and McManus). Corroborating these results, Ferreira et al. (1) showed that *Fasciola hepatica* extracts induced death of Chine Hamster Ovary (CHO) cells suggesting that some molecules contained in *F. hepatica* extracts could have a potential as a preventive or even curative anti-cancer substance. These data sustain the defensive shield of infections, as anticipated by the cancer hygiene hypothesis (2).

In the theme of helminths evolution, Laila A. Nahum and co-workers (Fundação Oswaldo Cruz (FIOCRUZ), Belo Horizonte, Brazil) submitted a mini-review discussing the evolutionary perspective of helminths and cancer (Scholte et al). Ruben Fernandes and colleagues (I3S, Instituto de Investigação e Inovação da Universidade do Porto, Porto, Portugal) commented how *Theileria* parasites maintain host leucocyte transformation through secretion of a prolyl isomerase (Fernandes et al).

Narong Khuntikeo and collaborators (Khon Kaen University, Khon Kaen, Thailand) presented a review concerning a brief update of the current situation regarding the natural history of opisthorchiasis and health burden of cholangiocarcinoma in Southeast Asia. This review also describes a comprehensive approach to tackling these issues being implemented in Thailand under the “Cholangiocarcinoma Screening and Care Program” (Khuntikeo et al). Francisco Almeida et al. (Centro Hospitalar São João, Porto, Portugal) filled a gap in the literature concerning the prevalence of *Fasciola hepatica* in the North of Portugal (Botelho et al). Finally, Michael H. Hsieh and Kenji Ishida (Biomedical Research Institute, Rockville, MD, United States) reviewed Urogenital Schistosomiasis-Related Bladder Cancer providing an update highlighting the most recent studies on schistosome-associated bladder cancer, including those that focus on identifying changes in host biology during *S. haematobium* infection (Ishida and Hsieh).

Feedback from Frontiers has been overwhelmingly positive, with many of the papers of this research topic having more the 1,000 views. We hope you will be informed by this e-book, as well as enjoy the authors' studious contributions, that the presented data and ideas will drive forward the field toward better control or even a cure for parasite-associated cancers, and that this Research Topic will promote investigations in parasite and related cancers.

## Author Contributions

All authors listed have made a substantial, direct and intellectual contribution to the work, and approved it for publication.

### Conflict of Interest Statement

The authors declare that the research was conducted in the absence of any commercial or financial relationships that could be construed as a potential conflict of interest.
